# Circulating bile acids predict outcome in critically ill patients

**DOI:** 10.1186/s13613-017-0272-7

**Published:** 2017-05-02

**Authors:** Thomas Horvatits, Andreas Drolz, Karoline Rutter, Kevin Roedl, Lies Langouche, Greet Van den Berghe, Günter Fauler, Brigitte Meyer, Martin Hülsmann, Gottfried Heinz, Michael Trauner, Valentin Fuhrmann

**Affiliations:** 1 Division of Gastroenterology and Hepatology, Department Internal Medicine 3, Medical University of Vienna, Vienna, Austria; 2 Department of Intensive Care Medicine, University Medical Center Hamburg-Eppendorf, Martinistraße 52, 20246 Hamburg, Germany; 3 Clinical Division and Laboratory of Intensive Care Medicine, Department of Cellular and Molecular Medicine, KU Leuven, Louvain, Belgium; 4 Clinical Institute of Medical and Chemical Laboratory Diagnostics, Medical University of Graz, Graz, Austria; 5 5th Medical Department, Kaiser Franz Josef Spital - SMZ Süd, Vienna, Austria; 6 Division of Cardiology, Department Internal Medicine 2, Medical University of Vienna, Vienna, Austria

**Keywords:** Bile acids, Cholestasis, Cardiogenic shock, Septic shock, Critically ill patients, ICU

## Abstract

**Background:**

Jaundice and cholestatic hepatic dysfunction are frequent findings in critically ill patients associated with increased mortality. Cholestasis in critically ill patients is closely associated with stimulation of pro-inflammatory cytokines resulting in impaired bile secretion and subsequent accumulation of bile acids.

Aim of this study was to evaluate the clinical role of circulating bile acids in critically ill patients.

**Methods:**

Total and individual serum bile acids were assessed via high-performance liquid chromatography in 320 critically ill patients and 19 controls.

**Results:**

Total serum bile acids were threefold higher in septic than cardiogenic shock patients and sixfold higher than in post-surgical patients or controls (*p* < 0.001). Elevated bile acid levels correlated with severity of illness, renal dysfunction and inflammation (*p* < 0.05). Total bile acids predicted 28-day mortality independently of sex, age, serum bilirubin and severity of illness (HR 1.041, 95% CI 1.013–1.071, *p* < 0.005). Best prediction of mortality of total bile acids was seen in patients suffering from septic shock.

**Conclusions:**

Individual and total BAs are elevated by various degrees in different shock conditions. BAs represent an early predictor of short-term survival in a mixed cohort of ICU patients and may serve as marker for early risk stratification in critically ill patients. Future studies should elucidate whether modulation of BA metabolism and signalling influences the clinical course and outcome in critically ill patients.

**Electronic supplementary material:**

The online version of this article (doi:10.1186/s13613-017-0272-7) contains supplementary material, which is available to authorized users.

## Background

Jaundice and cholestatic liver dysfunction are found in 10–20% of critically ill patients and are associated with markedly increased mortality [1–6]. Cholestasis in critically ill patients is associated with the presence of shock, sepsis, major surgery, hepatotoxicity of drugs and parenteral nutrition [4, 6–8]. While no universal definition of cholestasis has been established [1, 6, 7, 9, 10], the complexity of cholestasis, characterized by impaired bile formation and flow and subsequent bile acid (BA) retention, may not be sufficiently reflected by bilirubin [6, 11]. Although hyperbilirubinemia and accumulation of serum BAs are frequent findings in critically ill patients, underlying molecular pathways and clinical implications are still poorly understood. The clinical impact of serum BAs in critically ill patients has been assessed in a few studies focusing on sepsis [2, 6, 12, 13].

Cholestasis and sepsis are closely connected as expression of pro-inflammatory cytokines results in impaired bile secretion [1, 3, 13–15]. BAs are primarily synthesized in the liver out of cholesterol undergoing enterohepatic circulation (biliary excretion/ileal reabsorption) and then converted to secondary BAs by the gut microbiota [15–24] BA’s metabolic (regulation of lipid and energy homeostasis) and signalling properties are regulated via a complex network of nuclear receptors (NRs) such farnesoid-X-receptor (FXR) and G-protein-coupled BA receptor (TGR5) [17, 22–24]. BAs are directly vasoactive mediators and furthermore are capable of stimulating hepatocytes’ production of proinflammatory cytokines [25–29]. Furthermore, BAs directly influence cardiac function (myocardial contractility and relaxation) [30, 31]. Whether BA retention is a distinct pathophysiological entity or a biochemical epiphenomenon of severity of critical illness or even a compensatory mechanism with potential beneficial (e.g. metabolic or even anti-inflammatory) effects remains unclear [6].

Aim of this study was to assess the clinical relevance of circulating BAs in a large cohort of critically ill patients.

## Patients and methods

### Patients and serum analysis

Serum samples were taken from ICU patients (*n* = 320), enrolled in a prospective observational study performed at the University Hospital of Vienna [32]. For reasons of comparison, 19 controls undergoing elective restorative rectal surgery (matched to a general ICU population) were included [2]. The study was approved by the ethics committee of the Medical University of Vienna. Patients’ informed consent was obtained.

ICU patients were classified in four groups based on admission diagnoses (cardiogenic shock, septic shock, post-surgical admission and others). Inclusion criteria were ICU admission and age >18 years. Patients with liver cirrhosis and primary cholestatic disorders were excluded from the study.

Data collection was performed on a daily basis. Simplified Acute Physiology Score 2 (SAPS2) [33], as well as Acute Physiology And Chronic Health Evaluation Score 2 (APACHE2) [34], was calculated on admission. Clinical characteristics as well as 28-day mortality and ICU-survival were recorded.

### Bile acids

Serum BAs were measured in samples obtained from patients on admission and 48 h thereafter. BAs (as listed below) were assessed as unconjugated acids and taurine and glycine conjugates by tandem mass spectrometry. Free BAs and conjugates were detected by three multiple reaction monitoring (MRM) experiments, within one high-performance liquid chromatography (HPLC) run, due to variable ionization and fragment properties. HPLC was performed on a reversed phase (C_18_) column using a methanol–water gradient for chromatographic solution of isobaric BA. Quantification was done using deuterated internal standards and correlation of peak area ratios in linear regression.

TUDC (tauroursodeoxycholic acid); TC (taurocholic acid); TDC (taurodeoxycholic acid); TCDC (taurochenodeoxycholic acid); TLC (taurolithocholic acid); GCDC (glycochenodeoxycholic acid); GC (glycocholic acid); GDC (glycodeoxycholic acid); GUDC (glycoursodeoxycholic acid); GLC (glycolithocholic acid); UDC (ursodeoxycholic acid); CL (cholic acid); CDC (chenodeoxycholic acid); DC (deoxycholic acid); LC (lithocholic acid); and TBA (total bile acids) were determined.

Fasting TBAs range between 1.5 and 3.1 μmol/l [35, 36].

### Definitions

Cardiogenic shock was defined by (1) low systolic blood pressure (<90 mmHg) without use of inotropes/vasopressors, (2) decreased cardiac output assessed by any method, reduced mixed or central venous oxygen saturation, (3) absence of hypovolemia, (4) signs of organ malperfusion (oliguria, lactate-acidosis, cyanosis, centralization, changes in mental status) [37, 38].

Septic shock was defined as sepsis (suspected/present source of infection) and ≥2 systemic inflammatory response syndrome (SIRS) criteria: tachycardia (>90 beats per minute); tachypnea (>20 breaths/minute or partial pressure of carbon dioxide < 32 mmHg); temperature (>38.3/<36 °C); white blood cell count (>12/<4 × 10^9^/l) plus hypotension [39, 40].

### Management

Patients with cardiogenic or septic shock were treated according to standardized protocols [37, 39]. Intravenous fluid administration as well as vasopressor therapy was initiated in patients meeting shock criteria aiming to maintain a mean arterial blood pressure of >65 mmHg. Early initiation of broad-spectrum antibiotic treatment was performed according standardized protocols [39]. Antimicrobial therapy was adapted to culture results. Dialysis was performed in patients with renal failure and/or metabolic acidosis.

### Statistical analysis

Continuous variables were described as median and 25–75% interquartile range (IQR), and for categorical variables absolute and relative parameters were presented. Correlation analysis was performed using Spearman’s correlation. Continuous variables were compared using Mann–Whitney *U* test, and categorical variables were compared using Chi square tests. A forward stepwise procedure was used to identify most potent predictors of outcome variable. The overall diagnostic test accuracy of BAs was assessed by receiver operating characteristics expressed as their area under the receiver operating characteristics curve (AUROC). For data management and analyses, we used MS Excel 2008 for Mac (Microsoft Corp., Redmond, WA, USA), SPSS 17 for Mac (SPSS, Inc. Chicago, IL). All *p* values reported are two-sided, and *p* < 0.05 was considered as significant.

## Results

### Baseline characteristics

Three hundred and twenty ICU patients and 19 controls undergoing elective restorative rectal surgery were included in this prospective observational cohort-type study.

One hundred and forty-six (46%) patients were admitted due to cardiogenic shock, 56 patients (18%) following cardiothoracic surgery, 43 patients (13%) due to septic shock and 75 patients (23%) due to other diagnoses, such as non-infection related respiratory, neurological or haematological and/or oncological disorders. One hundred and seventeen patients were female (35%), and median age was 66 years (IQR 56–75). Median SAPS2-score was 49 (IQR 33–67), and median APACHE2-score was 21 (IQR 15–30).

Cardiogenic shock occurred due to myocardial infarction (38%), decompensation of congestive heart failure (24%), valvular heart disease (11%), and others (27%) such as pericardial effusion or myocarditis. Most common causes of sepsis were pneumonia (46%), urogenital tract infection (12%), catheter or blood stream infection (12%) and others (30%), such as endocarditis, meningitis or infectious complications following surgery.

Detailed patients’ characteristics are provided in Table 1.

**Table 1:** Patient characteristics

Parameter	Cardiogenic shock	Septic shock	Post-surgery	Others
n (%)	146 (46)	43 (13)	56 (18)	75 (23)
Female (%)	50 (34)	18 (42)	33 (39)	27 (36)
Age (years)	66 (57–73)	64 (60–73)	69 (56–77)	62 (52–76)
APACHE2	25 (17–34)	28 (21–36)	15 (10–19)	16 (12–26)
SAPS2	56 (41–77)	62 (44–74)	38 (27–51)	37 (27–53)
Vasopressor (%)^c, e, f^	146 (100)	43 (100)	53 (95)	31 (41)
Mechanical ventilation (%)^b, c, d, e, f^	92 (63)	26 (61)	47 (84)	23 (31)
RRT (%)^b, c, d^	34 (25)	10 (25)	3 (6)	6 (10)
28-day mortality (%)^b, c, d, e^	30 (21)	11 (26)	2 (4)	3 (4)
*Serum laboratory parameters*				
CRP (mg/dl)^a, b, d, e, f^	5 (1.5–8.3)	11.4 (4.5–24.1)	2 (0.6–5)	5 (2–8.3)
Creatinine (mg/dl)^b, c, d, e^	1.4 (0.9–2)	1.7 (1.1–3.4)	1 (0.8–1.2)	1.1 (0.9–1.6)
WBC (G/l)^a, e, f^	10.3 (7.6–13)	11.9 (8.5–19.6)	11.3 (8.8–15)	9.1 (7.5–11.8)
Fibrinogen (mg/dl)^a, b, d, e, f^	418 (318–529)	489 (417–647)	277 (216–387)	392 (246–479)
AST (U/l)^c^	58 (30–153)	41 (26–84)	50 (32–69)	30 (20–58)
Bilirubin (mg/dl)^b, c, d, e^	0.8 (0.5–1.2)	0.8 (0.6–1.3)	0.6 (0.5–0.8)	0.6 (0.5–0.9)

Data are shown as median and IQR

*RRT* renal replacement therapy

^a^ *p* < 0.05 cardiogenic shock versus septic shock

^b^ *p* < 0.05 cardiogenic shock versus post-surgery

^c^ *p* < 0.05 cardiogenic shock versus others

^d^ *p* < 0.05 septic shock versus post-surgery

^e^ *p* < 0.05 septic shock versus others

^f^ *p* < 0.05 post-surgery versus others

### Total serum bile acids and individual composition

TBAs on admission were significantly higher in ICU patients compared to controls (*p* < 0.05). TBAs were threefold higher in septic than cardiogenic shock patients and sixfold higher than in surgical patients and controls (*p* < 0.001) as shown in Table 2. TBAs, CL, GCDC, GC and bilirubin were significantly higher in cardiogenic shock patients compared to patients following surgery (*p* < 0.05). GCDC was threefold higher in septic compared to cardiogenic shock patients and ninefold higher than in those after cardiothoracic surgery (*p* < 0.05). Hyperbilirubinemia (serum bilirubin ≥ 2 mg within 48 h of admission) was observed more often in patients with septic shock (33%) compared to cardiogenic shock (21%) and patients after surgery (5%) (*p* < 0.05).

**Table 2:** Serum levels of individual bile acids in critically ill and control patients

Parameter	Cardiogenic shock	Septic shock	Post-surgery	Controls
TC^c, d, e, f^	0.15 (0.09–0.49)	0.25 (0.11–0.73)	0.12 (0.08–0.32)	0.02 (0.02–0.02)
TCDC^c, d, e, f^	0.27 (0.1–0.79)	0.5 (0.08–1.83)	0.11 (0.05–0.54)	0.01 (0.01–0.01)
TDC^d, e, f^	0.19 (0.03–0.49)	0.13 (0.01–0.52)	0.12 (0.03–0.52)	0.03 (0.03–0.03)
TLC^b, d, e, f^	0.07 (0.05–0.11)	0.06 (0.03–0.08)	0.05 (0.03–0.07)	0.01 (0.01–0.01)
TUDC^d, e^	0.09 (0.07–0.12)	0.11 (0.07–0.17)	0.08 (0.07–0.3)	0.01 (0.01–0.01)
GC^a, b, c, d, e, f^	0.35 (0.24–0.71)	0.61 (0.32–2.3)	0.26 (019–0.37)	0.03 (0.03–0.1)
GCDC^a, b, c, d, e, f^	0.34 (0.12–1.29)	0.96 (0.21–2.5)	0.11 (0.05–0.7)	0.05 (0.05–0.36)
GDC^d, e, f^	0.29 (0.07–0.58)	0.56 (0.11–1.03)	0.22 (0.06–0.97)	0.01 (0.01–0.01)
GLC	0.09 (0.06–0.19)	0.09 (0.07–0.13)	0.13 (0.11–0.15)	
GUDC^d, e, f^	0.06 (0.04–0.11)	0.09 (0.04–0.13)	0.05 (0.04–0.1)	0.02 (0.02–0.02)
UDC^d, e, f^	0.04 (0.03–0.07)	0.07 (0.02–0.13)	0.03 (0.02–0.14)	0.01 (0.01–0.07)
CL^b, c^	0.04 (0.03–0.05)	0.04 (0.03–0.17)	0.03 (0.02–0.04)	0.04 (0.01–0.05)
CDC	0.09 (0.05–0.29)	0.22 (0.05–0.9)	0.1 (0.06–0.21)	0.1 (0.09–0.15)
DC^d, e, f^	0.1 (0.08–0.18)	0.09 (0.07–0.25)	0.09 (0.07–0.17)	0.05 (0.05–0.08)
LC^b, d^	0.05 (0.04–0.06)	0.04 (0.03–0.06)	0.04 (0.03–0.05)	0.04 (0.04–0.04)
TBA^a, b, c, d, e^	1.18 (0.63–4.08)	4.28 (1.15–6.96)	0.68 (0.38–1.51)	0.62 (0.41–0.92)

Bile acids on admission are shown as median and IQR; bile acids (μmol/l)

*TC* taurocholic acid, *TCDC* taurochenodeoxycholic acid, *TDC* taurodeoxycholic acid, *TLC* taurolithocholic acid, *TUDC* tauroursodeoxycholic acid, *GCDC* glycochenodeoxycholic acid, *GC* glycocholic acid, *GDC* glycodeoxycholic acid, *GLC* glycolithocholic acid, *GUDC* glycoursodeoxycholic acid, *UDC* ursodeoxycholic acid, *CL* cholic acid, *CDC* chenodeoxycholic acid, *DC* deoxycholic acid, *LC* lithocholic acid, *TBA* total bile acids

^a^ *p* < 0.05 cardiogenic shock versus septic shock

^b^ *p* < 0.05 cardiogenic shock versus post-surgery

^c^ *p* < 0.05 septic shock versus post-surgery

^d^ *p* < 0.05 cardiogenic shock versus controls

^e^ *p* < 0.05 septic shock versus controls

^f^ *p* < 0.05 post-surgery versus controls

**Fig. 1:**
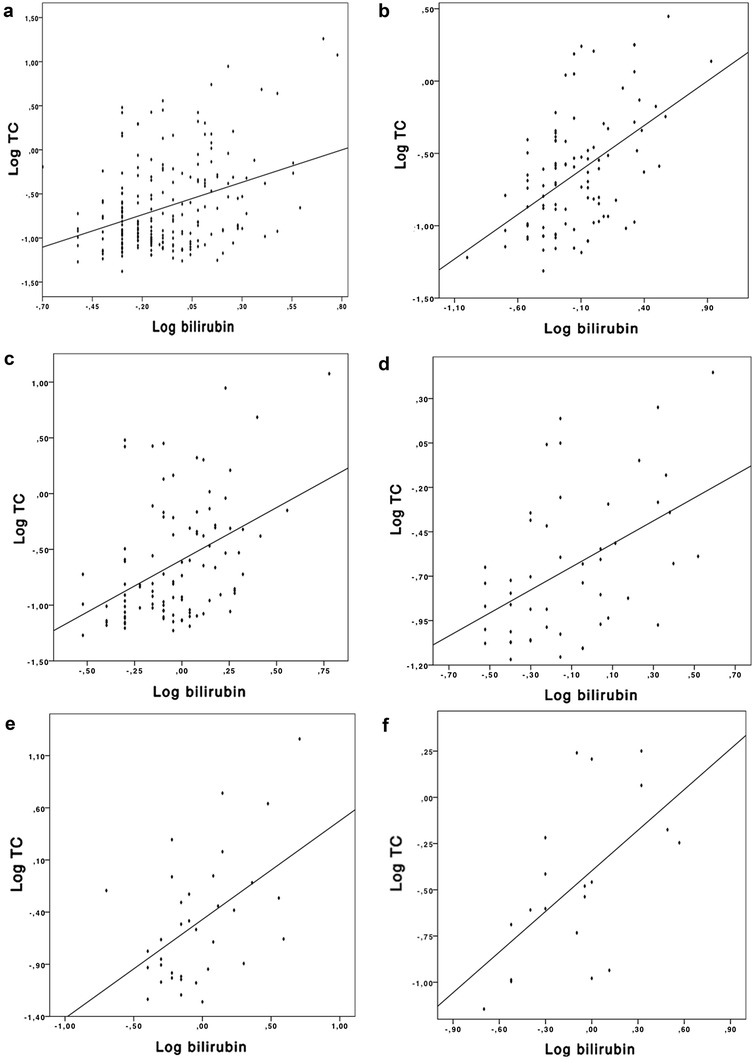
Correlation of serum bile acids and bilirubin. Correlation Log-TC and Log-bilirubin in all patients on admission (**a**); in all patients 48 h after admission (**b**); in cardiogenic shock patients on admission (**c**); in cardiogenic shock patients 48 h after admission (**d**); in septic patients on admission (**e**); in septic patients 48 h after admission (**f**)

On admission, individual and TBAs correlated with bilirubin in the overall cohort (TC, TCDC, TUDC, GCDC, GC, TBA) as shown in Additional file 1: Table S1. We observed a correlation of TUDC and TC and direct bilirubin (*r* 0.36, *p* = 0.001, *r* 0.32, *p* < 0.001). Best correlation was seen 48 h after admission (*p* < 0.05), as shown in Fig. 1.

Serum BAs (TC, TCDC, GC, GCDC, GLC, CL, TBA) correlated with APACHE2-score and SAPS2-score, as shown in Additional file 1: Table S3. Furthermore, GC and GDC correlated with arterial serum lactate (*r* 0.19, *p* < 0.005; *r* 0.22, *p* < 0.05). TCDC, TC, GC, LC and TBAs correlated inversely with mean arterial blood pressure (*r* −0.15, *p* < 0.05; *r* −0.18, *p* < 0.05; *r* −0.13, *p* < 0.05; *r* −0.16, *p* < 0.05; *r* −0.15, *p* < 0.01).

Individual and TBAs correlated with parameters of inflammation by means of CRP, white blood count and fibrinogen (*p* < 0.05), as illustrated in Additional file 1: Table S4.

Circulating BAs correlated significantly with markers of kidney function like creatinine and blood urea nitrogen (*p* < 0.05), as shown in Additional file 1: Table S5. Furthermore, patients undergoing renal replacement therapy had significantly higher levels of TC, GC and TBAs (*p* < 0.05).

### Bile acids in septic and cardiogenic shock

Individual as well as TBAs on admission sharply increased in patients with septic or cardiogenic shock compared to controls, as shown in Table 2.

Highest levels of TBAs were observed in patients with urosepsis, increasing by the half in comparison with catheter and blood stream infection and almost fourfold higher than in pneumonia. TCDC was 42-fold increased in patients with blood stream infection compared to those with pneumonia as source of septic shock (*p* < 0.05). In patients with cardiogenic shock, TBAs were threefold higher in decompensated heart failure compared to acute myocardial infarction (*p* < 0.001) and twofold higher than in valvular heart disease.

Individual and TBAs correlated with serum bilirubin levels in cardiogenic and septic shock on admission and 48 h thereafter as shown in Additional file 1: Table S2. Best correlation with serum bilirubin was seen in septic shock 48 h after admission, TC (*r* 0.6, *p* = 0.005), as illustrated in Fig. 1.

In cardiogenic shock, circulating BAs correlated significantly with arterial serum lactate (GC: *r* 0.31, *p* < 0.005) and pro-BNP levels (TC: *r* 0.62, *p* < 0.05; GCDC: *r* 0.66, *p* = 0.005; GUDC: *r* 0.683, *p* < 005; CL: *r* 0.58, *p* < 0.05; TBA: *r* 0.59, *p* < 0.05). There was no significant correlation of BAs and cardiac enzymes, such as myocardial muscle creatine kinase and troponin T (*p* = n.s.). GLC was significantly elevated in patients undergoing cardiopulmonary resuscitation (*p* < 0.05).

### Bile acids and 28-day mortality

TBAs, individual GCDC, GC and serum bilirubin on admission were significantly higher in 28-day non-survivors (*p* < 0.05).

**Fig. 2:**
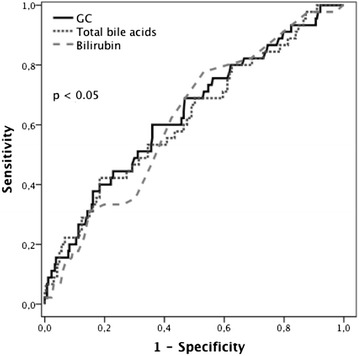
Total and individual serum bile acids predicting 28-day mortality. ROC analysis for prediction of 28-day mortality of TBA (total bile acids), GC (glycocholic acid) and serum bilirubin on admission. AUROC of GC: 0.64, *p* = 0.005; TBA: 0.62, *p* < 0.05; bilirubin: 0.61, *p* < 0.05

TBAs on admission predicted 28-day mortality with an AUROC of 0.63 (*p* < 0.01) followed by bilirubin (0.62, *p* < 0.05), GCDC (0.6, *p* < 0.05) and delta TBAs (as difference from baseline to 48 h) (0.55, *p* < 0.05). Best prediction of 28-day mortality was seen in GC (AUROC 0.64, *p* < 0.005), as illustrated in Fig. 2.

Univariate cox hazard regression showed a significant association of baseline TBAs with 28-day mortality (HR per increase of TBAs 1 μmol/l 1.033, 95% CI 1.018–1.049, *p* < 0.001). TBAs remained associated with 28-day mortality after correction for sex, age, bilirubin and severity of critical illness via APACHE2 score (HR 1.041, 95% CI 1.013–1.071, *p* < 0.005), as show in Table 3.

**Table 3:** Cox hazard regression analysis of baseline TBA and bilirubin associated with 28-day mortality

Parameter	HR (95% CI)	*p* value
TBA	1.033 (1.018–1.049)	<0.001
TBA (age)^a^	1.033 (1.017–1.049)	<0.001
TBA (age, sex)^a^	1.033 (1.017–1.049)	<0.001
TBA (age, sex, bilirubin)^a^	1.044 (1.014–1.075)	<0.005
TBA (age, sex, bilirubin, APACHE2)^a^	1.041 (1.013–1.071)	<0.005

*CI* confidence interval

^a^Corrected for covariates in brackets, HR, hazard ratio per increase of TBA 1 μmol/l or bilirubin 1 mg/dl

**Fig. 3:**
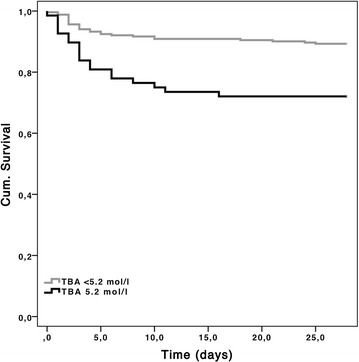
Kaplan–Meier’s plot of 28-day survival. 28-day survival rate was significantly lower in patients with TBA (total bile acids) on admission ≥ 5.2 μmol/l compared to patients < 5.2 μmol/l (72 vs. 89%, *p* < 0.001)

Youden index revealed TBAs on admission ≥5.2 μmol/l as best cut-off for discriminating between survivors and non-survivors. 28-day survival rate was significantly lower in patients with TBAs ≥5.2 μmol/l compared to TBAs < 5.2 μmol/l (72 vs. 89%, *p* < 0.001). Kaplan–Meier’s plot of 28-day survival is shown in Fig. 3.

An increase of delta TBAs (as difference from baseline to 48 h) of ≥10 µmol/l was associated with significantly higher mortality rates (50 vs. 13%, *p* < 0.05).

## Discussion

Early hepatic dysfunction is a frequent finding in critically ill patients [6, 7]. Prolonged length of stay as well as worse outcome has been reported to be strongly associated with cholestatic impairment [1, 3, 5, 6]. However, there is lack of data regarding the clinical relevance of early hepatic dysfunction by means of altered serum BA levels and composition in different disease entities in critically patients. Therefore, we aimed to describe the clinical impact of circulating serum BAs and individual composition in a cohort of critically ill patients, mainly suffering from cardiogenic and septic shock.

Our results demonstrate an elevation of individual as well as TBAs in a heterogeneous group of critically ill patients compared to controls. Notably, the highest levels of BAs were found in patients with septic shock. In patients with cardio-circulatory disorders, serum BA levels were still significantly higher than in patients following cardiothoracic surgery or controls. Severity of underlying disease did not differ in patients with septic or cardiogenic shock or those following surgery or others. Potentially toxic conjugates of unconjugated primary BAs CL and CDC were highest in accordance with previous reports [2]. The lack of difference of primary BAs (e.g. CDC) in between groups may reflect the organisms’ ability of enhanced conjugation of probably toxic BAs under condition of critical illness. However, both increasing conjugated and unconjugated BAs during critical illness have been reported [3, 6]. BAs are known for cytotoxic effects, in particular hydrophobic BAs are capable of inducing irreversible cell damage [41]. A change in hydrophilic-hydrophobic balance towards hydrophobicity in ICU patients compared to controls may be assumed. In our cohort, highly hydrophobic LC and DC were significantly higher in patients with cardiogenic shock than in controls. Best correlation of BAs and severity of disease (SAPS2 score) was seen in glycine conjugates of toxic LC. Additionally, we observed a significant correlation of GC and GDC with arterial serum lactate levels.

BAs correlated with markers of inflammation like C-reactive protein, leucocytes and fibrinogen. Hydrophobic taurine conjugates of CDC were more than 40-fold higher in blood stream infection compared to pneumonia. The highest TBA levels were seen in urosepsis, followed by catheter and blood stream infection and pneumonia. Common pathogens of urosepsis as well as hospital-acquired pneumonia are gram-negative bacteria (e.g. *Escherichia coli, Pseudomonas aeruginosa, Haemophilus influenzae*) which are known to predominantly repress hepatobiliary transporter expression and subsequently BA retention in vivo [13, 15, 42, 43]. BAs are important signalling molecules interacting between liver, bile and gut and are involved in lipid and energy homeostasis [17]. They are a major regulator of the gut microbiota and directly interact with gut bacteria. It has been suggested that intestinal microorganisms benefit from metabolizing BAs as they acquire glycine/taurine [44]. Elevated intake of BAs results in changes of the gut microbiota by means of inhibition of *Actinobacteria* and *Bacteroidetes* [22, 44]. Altered gut integrity due to increased intestinal permeability and bacterial translocation represent important triggers of sepsis and sepsis-related organ dysfunction [45]. Serum BA levels are able to directly trigger inflammatory processes via cytokine expression [25–27]. Conversely, BA also have anti-inflammatory and immunosuppressive properties mediated by FXR and TGR5 via modulating anti-inflammatory gene expression [17, 46, 47] Thus, BAs may have both pro- and anti-inflammatory actions depending on the time course and concentrations of serum BA levels during sepsis [48].

Sepsis-related volume disturbances and subsequent hemodynamic changes seem to be relevant triggers of multifactorial genesis of renal failure in critically ill patients with cholestatic dysfunction [5]. Interestingly, we found a clear association of serum BA retention and need of RRT as well as serum markers of kidney dysfunction. Oxidative stress has been postulated as central mechanism inducing cholestasis associated renal impairment [49]. Recently, BAs have been implicated in kidney injury due to longstanding jaundice. Cholemic nephropathy represents typical histopathological alterations including tubular epithelial cell injury and intratubular cast formation in patients with cholestasis [50, 51]. In addition, solubility of BAs is increased in acidic milieu such as metabolic acidosis, which may reflect another factor for urinary cast formation and interstitial tubular damage [52].

BAs have directly vasoactive properties, inducing peripheral vasodilation via relaxing smooth muscle cells. Furthermore, alterations of BA metabolism have been found in cardiac disease and heart failure [30, 31, 53]. Recently, G-protein-coupled BA receptor-1 has been identified as central pathway in BA-mediated hemodynamic alterations [28, 29]. We observed a significant correlation of TC and TCDC with arterial blood pressure in our cohort. Valvular heart disease and subsequent congestive heart failure, chronic cardiac disorders, frequently lead to backward right ventricular heart failure with consecutive congestive hepatopathy and concomitant jaundice [5, 54]. Bilirubin and low serum albumin levels have been reported to be associated with elevated mortality rates in patients hospitalized for heart failure [55]. BAs impact on cardiac function as they modulate myocardial contractility and relaxation [30, 31]. They reduce duration of ventricular myocytes’ action potential and have proarrhythmogenic properties [30]. In our cohort, TBAs were significantly higher critically ill patients with cardiogenic shock compared to surgical patients and controls. Highest levels of serum BAs were seen in valvular heart disease and decompensated heart failure. Individual and TBAs correlated significantly with pro-BNP levels.

Serum BAs correlated with bilirubin in our heterogenous collective of critically ill patients. Strongest correlation was seen in septic patients, which even increased during course of time. The underlying mechanism of hyperbilirubinemia in critically ill patients is not fully understood. Whether cholestasis during critical illness is merely a biological epiphenomenon of the failing organ or even has protective effects remains unclear [2, 6, 56]. However, several factors influence bilirubin levels, such as infections of the liver, transfusion of red blood cells and/or hemolysis [6]. Therefore, bilirubin may not represent the ideal parameter for assessing hepatic organ dysfunction in the ICU [6]. In our cohort, both BAs and bilirubin were elevated in patients that died within the first 28 days. However, serum BAs showed better predictive properties than bilirubin.

Our data demonstrated that an early increase of circulating BAs in a heterogeneous collective of critically ill patients is associated with increased 28-day mortality. TBAs predicted short-term mortality independently of serum bilirubin levels and severity of critical illness. BA’s predictive properties seem to be higher than bilirubin’s in accordance with another recent publication [3]. Best prediction of mortality was observed in patients with septic shock. As TBAs can easily be measured in standard hospital laboratories, available at manageable costs BAs could represent an early non-invasive marker for predicting outcome and serve as a new tool for early risk stratification in critically ill patients.

Apart from its prognostic implications, future studies should clarify if modulation of BA levels has clinical implications on new onset of organ failure and outcome. Advanced dialysis systems like liver support devices are capable of eliminating albumin bound substances (such as serum BAs) [57, 58]. Furthermore, several drugs can modify BA levels and signalling. BA sequestrants such as non-absorbed resins lower BA levels via binding and removing them from enterohepatic circulation [16]. Intestinal interception in BA transport and signalling may prevent systemic side effects while restoring BA homeostasis and gut integrity [47, 59, 60]. Future studies should clarify whether device-based or medication-based BA modifications including ligands for FXR and TGR5 may have an impact on clinical course and outcome of critically ill patients.

## Conclusion

To conclude, our study demonstrates that circulating individual and total BAs are elevated by various degrees in different shock conditions. BAs represent an early predictor of short-term survival in a mixed cohort of ICU patients and may serve as a novel marker for early risk stratification in critically ill patients. Future studies should elucidate whether modulating BA metabolism or elimination of BAs by extracorporeal devices influences clinical course and outcome.

## Additional file

**Additional file 1: Table S1.** Correlation of serum bile acids and bilirubin in all patients. **Table S2.** Correlation of serum bile acids and bilirubin in septic and cardiogenic shock. **Table S3.** Correlation of serum bile acids severity of critical illness on admission. **Table S4.** Correlation of serum bile acids on admission and marker of kidney function. **Table S5.** Correlation of serum bile acids and inflammation parameters on admission.

## Abbreviations

BA: bile acid
TBA: total bile acid
NRs: nuclear receptors
FXR: farnesoid-X-receptor
TGR5: G-protein-coupled BA receptor
ICU: intensive care unit
SAPS2: Simplified Acute Physiology Score 2
APACHE2: Acute Physiology And Chronic Health Evaluation Score 2
MRM: multiple reaction monitoring
mmHg: millimeter per mercury
HPLC: high-performance liquid chromatography
SIRS: systemic inflammatory response syndrome
IQR: interquartile range
AUROC: area under the receiver operating characteristics curve
