# Supplementary material for: Circulating bile acids predict outcome in critically ill patients
Source: Ann Intensive Care. 2017 May 2;7:48. doi: 10.1186/s13613-017-0272-7 (PMC5413465; doi:10.1186/s13613-017-0272-7)
Supplement: Supplementary file 1 — Additional file 1: Table S1. Correlation of serum bile acids and bilirubin in all patients. Table S2. Correlation of serum bile acids and bilirubin in septic and cardiogenic shock. Table S3. Correlation of serum bile acids severity of critical illness on admission. Table S4. Correlation of serum bile acids on admission and marker of kidney function. Table S5. Correlation of serum bile acids and inflammation parameters on admission. [file 13613_2017_272_MOESM1_ESM.docx]

**Table S1:**

Correlation of serum bile acids and bilirubin in all patients

| Correlation with bilirubin | admission | 48 hours after admission |
| --- | --- | --- |
| TC | r: 0.28, p < 0.001 | r: 0.46, p < 0.001 |
| TCDC | r: 0.27, p < 0.001 | r: 0.06, p = 0.668 |
| TDC | r: 0.14, p = 0.15 | r: 0.28, p < 0.005 |
| TUDC | r: 0.28, p < 0.05 | r: 0.26, p = 0.111 |
| GC | r: 0.2, p < 0.001 | r: 0.37, p < 0.001 |
| GCDC | r: 0.18, p < 0.005 | r: 0.25, p < 0.005 |
| GDC | r: 0.1, p = 0.26 | r: 0.27, p < 0.05 |
| TBA | r: 0.17, p < 0.005 | r: 0.29, p = 0.001 |

Spearman’s correlation of serum bile acids and bilirubin on admission and 48 hours thereafter.

**Table S2:**

Correlation of serum bile acids and bilirubin in septic and cardiogenic shock

| Correlation of BA with bilirubin |  |
| --- | --- |
| *Cardiogenic shock* | *on admission* |
| TC | r: 0.4, p < 0.001 |
| TCDC | r: 0.34, p = 0.001 |
| TUDC | r: 0.43, p < 0.05 |
| GC | r: 0.25, p < 0.005 |
| GCDC | r: 0.27, p < 0.005 |
| TBA | r: 0.27, p = 0.001 |
| *Cardiogenic shock* | *48 hours after admission* |
| TC | r: 0.46, p = 0.001 |
| GC | r: 0.35, p = 0.005 |
| *Septic shock* | *on admission* |
| TC | r: 0.48, p < 0.005 |
| TCDC | r: 0.49, p < 0.005 |
| *Septic shock* | *48 hours after admission* |
| TC | r: 0.6, p =0.005 |
| TDC | r: 0.46, p < 0.05 |

Spearman’s correlation of serum bile acids and bilirubin on admission and 48 hours thereafter.

**Table S3:**

Correlation of serum bile acids severity of critical illness on admission

|  | APACHE2 | SAPS2 |
| --- | --- | --- |
| TC | r: 0.21, p < 0.005 | r: 0.18, p < 0.01 |
| TCDC | r: 0.19, p < 0.05 | r: 0.15, p < 0.05 |
| GC | r: 0.24, p < 0.001 | r: 0.19, p = 0.001 |
| GCDC | r: 0.18, p < 0.005 | r: 0.13, p < 0.05 |
| GLC | r: 0.29, p = 0.091 | r: 0.34, p < 0.05 |
| CL | r: 0.17, p < 0.05 | r: 0.13, p = 0.05 |
| TBA | r: 0.25, p < 0.001 | r: 0.18, p = 0.001 |

Spearman’s correlation of serum bile acids and severity of critical illness assessed via APACHE-2 and SAPS-2 score.

**Table S4:**

Correlation of serum bile acids on admission and marker of kidney function

|  | creatinine | BUN |
| --- | --- | --- |
| TC | r: 0.22, p = 0.001 | r: 0.29, p < 0.001 |
| TCDC | r: 0.21, p < 0.005 | r: 0.29, p < 0.001 |
| TDC | r: 0.28, p < 0.005 | r: 0.32, p = 0.001 |
| TUDC | r: 0.35, p < 0.005 | r: 0.38, p = 0.001 |
| TLC | r: 0.24, p = 0.093 | r: 0.31, p < 0.05 |
| GC | r: 0.27, p < 0.001 | r: 0.25, p < 0.001 |
| GCDC | r: 0.22, p < 0.001 | r: 0.27, p < 0.001 |
| GDC | r: 0.37, p < 0.001 | r: 0.25, p = 0.005 |
| GUDC | r: 0.16, p < 0.05 | r: 0.14, p = 0.065 |
| CL | r: 0.21, p < 0.005 | r: 0.22, p = 0.001 |
| DC | r: 0.22, p < 0.005 | r: 0.17, p < 0.05 |
| TBA | r: 0.29, p < 0.001 | r: 0.31, p < 0.001 |

Spearman’s correlation of serum bile acids and creatinine and blood urea nitrogen

**Table S5:**

Correlation of serum bile acids and inflammation parameters on admission

|  | TC | GC | TBA |  |
| --- | --- | --- | --- | --- |
| CRP | r: 0.16, p < 0.05 | r: 0.16, p = 0.005 | r: 0.14, p < 0.05 |  |
|  |  |  |  |  |
|  | TCDC | TUDC | GCDC | TBA |
| WBC | r: -0.18, p < 0.05 | r: - 0.23, p < 0.05 | r: - 0.14, p < 0.05 | r: - 0.13, p < 0.05 |
|  |  |  |  |  |
|  | GC | GCDC | CDC | TBA |
| Fibrinogen | r: 0.14, p < 0.05 | r: 0.12, p < 0.05 | r: 0.19, p < 0.05 | r: 0.19, p < 0.05 |

Spearman’s correlation of serum bile acids and c-reactive protein (CRP), white blood count (WBC) and acute phase protein fibrinogen.
